# Microsatellite markers of the major histocompatibility complex genomic region of domestic camels

**DOI:** 10.3389/fgene.2022.1015288

**Published:** 2022-10-24

**Authors:** Ales Knoll, Jan Wijacki, Martin Plasil, Pamela A. Burger, Petr Horin

**Affiliations:** ^1^ Department of Animal Morphology, Physiology and Genetics, Faculty of Agronomy, Mendel University in Brno, Brno, Czechia; ^2^ Department of Animal Genetics, Faculty of Veterinary Medicine, University of Veterinary Sciences Brno, Brno, Czechia; ^3^ CEITEC-VETUNI, University of Veterinary Sciences Brno, Brno, Czechia; ^4^ Research Institute of Wildlife Ecology, University of Veterinary Medicine Vienna, Vienna, Austria

**Keywords:** genetic diversity, camels, microsatellite markers, major histocompatibility complex, *Camelus dromedarius*, *Camelus bactrianus*

## Abstract

We identified and characterized 11 polymorphic microsatellite markers suitable for routine testing (three in the MHC class I sub-region, four in MHC class II and four in the MHC class III sub-region) of dromedaries and Bactrian camels. In total, 38 dromedaries and 33 Bactrian camels were genotyped, and interspecific differences were observed in the numbers of alleles and in allelic frequencies, as well as in the observed heterozygosity. These loci may be used as markers to study the adaptive genetic diversity of the MHC region in Old World camels.

## Introduction

Domesticated camels, the dromedary (*Camelus dromedarius*) and the Bactrian camel (*Camelus bactrianus*), are economically and culturally important species in many countries worldwide. They are used for transportation and as a source of food. Camel milk and meat are important and valuable sources of protein (Ali et al., 2019). Since their domestication five or six thousand years ago, they have adapted to extreme environmental conditions, especially those of arid countries. Camels seem to be more resistant to some pathogens compared to other sympatric species (Burger 2016). Their immune system is characterized by several specific features, which makes them an informative model for immunological and immunogenetic studies (Ciccarese et al., 2019; Hussen and Schuberth 2021). Recent camel genome assemblies made possible the characterization of the camel immunogenome, its genetic diversity and its associations with important infections (Elbers et al., 2019; Lado et al., 2020; Ming et al., 2020a; Lado et al., 2021, 2022).

The molecules encoded by the Major Histocompatibility Complex, along with two genomic regions coding for Natural Killer cell receptors (NKRs), the Leukocyte Cell Receptor Complex (LRC) and the Natural Killer Cell Complex (NKC), are important parts of the mammalian immune system (Carrillo-Bustamante et al., 2016). Both of these types of genes are highly polymorphic, and their polymorphism is of adaptive value (Axtner and Sommer 2007). The multiple roles of the MHC in immunity and disease in humans and various mammalian species have been repeatedly reviewed (Traherne 2008). At the population level, an extensive genetic diversity of MHC genes has been reported in many mammalian species (Yuhki and O’Brien 1990; Kamath and Getz 2012). Often, this diversity is associated with important diseases (Shiina et al., 2009).

The MHC and NKR genomic regions of camelid species have been characterized only recently (Plasil et al., 2019a a,b; Futas et al., 2019). The genomic structure of the camelid MHC is similar to other mammalian species (Plasil et al., 2019a). However, the diversity of MHC genes seems to be lower than in most other species (Plasil et al., 2019b). Due to the existence of multiple highly similar loci, individual genotyping of MHC and NKR genes is a challenging task. Therefore, only limited information on the genetic diversity of MHC genes in various camel populations is available.

Genotyping microsatellite markers is an inexpensive and relatively easy method of assessing the genetic diversity of the MHC genomic regions in various animal species. MHC-linked microsatellite markers have been identified and some of them have been used for characterizing MHC polymorphism in various species, including domestic animals such as horses (Sommer 2005; Horecky et al., 2018; Jaworska et al., 2020). Microsatellite loci distributed throughout the genomes of dromedaries have been identified (Khalkhali-Evrigh et al., 2019). However, no detailed information on microsatellite loci mapping to MHC class I, II and III sub-regions is currently available. Recently, we have developed a panel of microsatellite markers for assessing the genetic diversity of NKR genomic regions in camels (Futas et al., 2019). Here, we present a set of MHC-linked microsatellites that can be used as markers of MHC population diversity and for association analyses.

The primary purpose of this work was to study the non-neutral, i.e., adaptive genetic diversity of camel populations. For this purpose, the MHC genomic region is a prime candidate, due to its crucial role in immune responses. The specific objective of this study was therefore to identify polymorphic microsatellite loci located within the MHC region in the two species of domestic camels, the dromedary (*Camelus dromedarius*) and the Bactrian camel (*Camelus bactrianus*)*.*

## Materials and methods

### Camels

DNA samples of 38 dromedaries (*Camelus dromedarius*) and 33 Bactrian camels (*Camelus bactrianus*) were analyzed. The dromedary hair samples were obtained from North African and Arabian countries during previous projects (Austrian Science Foundation (FWF) P1084-B17 and P24706-B25), and DNA extractions followed a simple salting-out protocol (De Volo et al., 2008). The Bactrian samples were collected in Mongolia for the purposes of another project and shared by prof. D. Modry, Vetuni Brno, Czechia.

All samples were collected in agreement with all legal and ethical standards by licensed veterinarians for the purposes of other projects. No sample analyzed in this study was collected specifically for the purposes of this particular project.

### Genomic resources

Genomic sequences of *Camelus dromedarius* from the CamDro2 whole genome assembly (https://www.ncbi.nlm.nih.gov/assembly/GCA_000803125.2/) NW_011591952.1 (3.29 Mb), NW_011591249.1 (0.33 Mb) and NW_011591121.1 (1.05 Mb) were screened to detect microsatellites and for primer design.

### Microsatellite detection and characterization

The search for microsatellites was conducted using the online microsatellite repeat finder WebSat (http://www.wsmartins.net/websat/) within the sequences NW_011591121.1 (Wu et al., 2014), NW_011591952.1 (Wu et al., 2014) and NW_011591249.1 (Wu et al., 2014).

Specific primers for amplifying the selected 32 microsatellites were designed *de novo* using the OLIGO software v4 and verified in the Primer Express v2.0 software (Applied Biosystems, Inc, Foster City, CA, United States). Aiming to optimize multiplex PCR protocols, all primers were designed using the same parameters (Ta, Tm, number of nucleotides). The forward primers were labelled by fluorescent dyes (6-FAM, HEX, NED and PET; Table 1). In addition, information on primers amplifying another six loci was obtained from the Baker Institute for Animal Health, Cornell University, Ithaca, NY (D. Antczak, D. Miller). They were also located within the sequences NW_011591121.1 (Wu et al., 2014), NW_011591952.1 (Wu et al., 2014) and NW_011591249.1 (Wu et al., 2014).

**TABLE 1:** Panel of markers selected for assessing the MHC diversity in camels.

Region	Marker	Primer name	Location in CamDro2	Primer sequence 5'—3′	Fluorescent dye (5′)	PCR mix	Locus database Id	Repeated sequence motif
MHC I	*CAM01*	CAMf_I_01	317 417	F: GCA GCA GAG ATA TGC ACA GAA T	6-FAM	A	NW_011591121.1	(TG)_n_
				R: CTG GCT TAC TTC ACT TGG AAT G				
MHC I	*CAM02*	1121_I_11	595 103	F: ACT GGG TCA GAA ACA CCA AG	6-FAM	C	NW_011591121.1	(AC)_n_
				R: CTG TGA GGG AGC TGT CTG TC				
MHC I	*CAM03*	CAMf_I_45	333 420	F: AAC CCT TCC TTC TGA GTG CTT GC	NED	A	NW_011591121.1	(TC)_n_
				R: GGG AAT GAG AGA GAC ACA GGG AAA				
MHC II	*CAM04*	CAMf_II_35	3 024 151	F: GCT GCT CTC CTT TCT GCT CTA C	6-FAM	B	NW_011591952.1	(AC)_n_
				R: GGT TAA GTG GGT GAG GCT ACA G				
MHC II	*CAM05*	CAMf_II_47	3 054 949	F: CCA GCT GCC CTT TGA GTC AAT C	HEX	B	NW_011591952.1	(AC)_n_
				R: CCC ACC CCG CAA TGT TAA AAT				
MHC II	*CAM06*	1592_II_07	3 219 385	F: TTG TTC CAG TGA TGG TGA TG	NED	C	NW_011591952.1	(GT)_n_
				R: ATG CTG AGG AGA GAC CAG AG				
MHC II	*CAM07*	1592_II_08	3 257 534	F: TTT CAA AAT CCT GCC AAC AC	NED	C	NW_011591952.1	(TG)_n_
				R: TTG TAC CCT GAG GTC TGC TC				
MHC III	*CAM08*	CAMf_III_26	2 742	F: AGA AGC AGT GGT GTG AGA TCC T	PET	A	NW_011591249.1	(GT)_n_
				R: TCA ATT TGA ACC ACA TGG ATG T				
MHC III	*CAM09*	CAMf_III_41	190 586	F: TTC AGA CAA CGT CAG CAT CTC T	NED	B	NW_011591249.1	(AC)_n_
				R: AGC CAG TGA GTG TAG GGA GTG T				
MHC III	*CAM10*	CAMf_III_42	232 193	F: CAG TTC CCC GCT GTA TAA TCT C	NED	B	NW_011591249.1	(GT)_n_
				R: CAG TGT CTA ACG TGG GTT TTG A				
MHC III	*CAM11*	1121_III_02	140 350	F: TGG CTT TAC ACA CAC ACA CG	NED	C	NW_011591249.1	(TG)_n_
				R: CCT GAA GTT TGA CCA GTT CG				

Based on the selection of primer pairs, protocols allowing three multiplex amplifications and paralleled detection of alleles were optimized. Each PCR was performed in a final volume of 10 µL. The PCR reaction mix consisted of 1.0 μL 10 × Taq Buffer (Top-Bio, ltd, Prague, Czech Republic), 0.5 U CombiTaq DNA polymerase (Top-Bio, ltd, Prague, Czech Republic), 200 µm each dNTP (Thermo Fisher Scientific Inc, Waltham, United States), 0.1 μL of each primer of concentration 10 μm, and 50 ng of genomic DNA. The volume of the reaction mix was adjusted to 10.0 μL with PCR H_2_O (Top-Bio, ltd, Prague, Czech Republic). Fragments were amplified in three separate PCR mixes: A, B and C. Mix A contained primers for markers *CAM001*, *CAM03* and *CAM08*. Mix B contained primers for amplification of microsatellites *CAM04*, *CAM05*, *CAM09* and *CAM10*. Markers *CAM02*, *CAM06*, *CAM07* and *CAM11* were amplified by mix C (Table 1).

All PCRs were performed in the thermal cycler ABI Verity 96 Well (Applied Biosystems Inc, Foster City, CA, United States) with the following protocol: initial denaturation at 95°C for 3 min; 30 cycles of denaturation at 95°C for 30 s, annealing at 60°C (mix A) and 58°C (mix B,C) for 30 s and elongation at 72°C for 30 s; final elongation at 72°C for 60 min and holding at 7°C.

All three sets of markers were then tested by fluorescent fragment analysis using an ABI PRISM 3500 Genetic Analyzer and sized with LIZ500 dye size standard (Life Technologies, Corp, Carlsbad, United States). The data obtained were analyzed using the GeneMapper software v4.1 (Life Technologies, Corp, Carlsbad, United States).

The dromedary primers were then used for PCR amplifications of Bactrian camel DNA samples.

### Validation of the microsatellite markers

The specificity of all selected markers (including flanking regions) was verified based on at least two independent PCRs using Sanger sequencing. The Mendelian inheritance of all markers was assessed in seven trios (father, mother, offspring) of *Camelus bactrianus* (Table 2).

**TABLE 2:** Example of the Mendelian inheritance of all markers in *Camelus bactrianus* in two trios.

	*CAM01*		*CAM02*		*CAM03*		*CAM04*		*CAM05*		*CAM06*		*CAM07*		*CAM08*		*CAM09*		*CAM10*		*CAM11*	
*father1*	370	374	302	304	194	196	340	340	284	294	147	149	296	302	374	374	288	290	376	400	250	258
*mother1*	366	382	304	306	194	196	340	340	274	284	143	149	296	296	374	374	288	292	376	396	246	252
*offspring*	370	382	304	304	194	196	340	340	284	294	143	149	296	296	374	374	288	292	376	396	252	258

	*CAM01*		*CAM02*		*CAM03*		*CAM04*		*CAM05*		*CAM06*		*CAM07*		*CAM08*		*CAM09*		*CAM10*		*CAM11*	
*father2*	366	376	306	320	194	196	340	346	274	288	145	149	296	296	374	374	288	304	376	406	246	246
*mother2*	364	372	314	324	194	196	346	346	284	284	141	147	294	302	374	374	288	288	376	418	242	246
*offspring*	364	366	306	314	196	196	340	346	274	284	147	149	296	302	374	374	288	288	376	376	246	246

### Characterization of the markers on a panel of camels

The microsatellite Toolkit for Excel (Park 2001) was used to calculate the major characteristics of molecular diversity for all selected markers genotyped in the panel of camels. Haplotype analysis was performed using Arlequin ver 3.5 (Excoffier and Lischer, 2010).

## Results

One hundred and twenty-two dinucleotide microsatellites were detected in all three MHC subregions (58 within the sequence NW_011591121.1, 31 in NW_011591952.1, and 33 in NW_011591249.1). Based on our previous experience, dinucleotide microsatellites with CA/TG motifs with perfect repetitions and at least seven motif repeats were preferred. Thirty-eight microsatellites (including six microsatellites from the Baker Institute for Animal Health) complied with the previously defined criteria. These microsatellites were tested in our set of animals. Even in the initial selection of microsatellites, no markers polymorphic in only one species were found. Some microsatellites were non-polymorphic (in both species) and were immediately removed from further analysis. Only 11 microsatellites were polymorphic and suitable for further characterization and routine testing (three in the MHC class I sub-region, four in MHC class II and four in the MHC class III sub-region).

**FIGURE 1:**
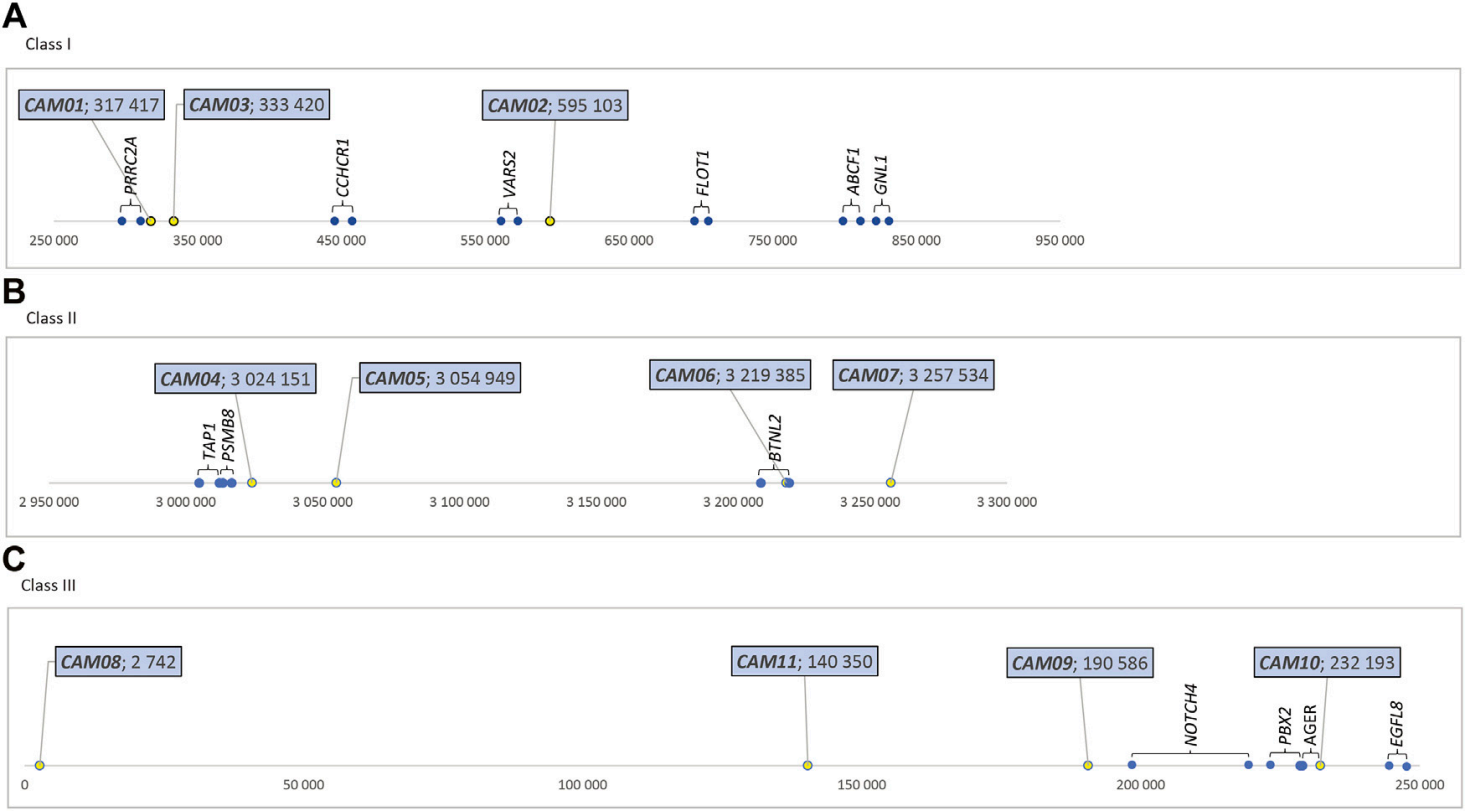
Relative positions of MHC-linked microsatellite markers (in bp), dromedary camel genome **(A)** Class I - in the sequence NW_011591121.1, https://www.ncbi.nlm.nih.gov/nuccore/NW_011591121.1?report=graph. **(B)** Class II - in the sequence NW_011591952.1, https://www.ncbi.nlm.nih. gov/nuccore/NW_011591952.1?report=graph **(C)** Class III - in the sequence NW_011591249.1, https://www.ncbi.nlm.nih.gov/nuccore/NW_011591249.1?report=graph.

The physical location of these 11 markers within the MHC region of dromedaries is shown in Figure 1. Their molecular characteristics are summarized in Table 1. A comparison between dromedaries and Bactrian camels is available in Table 3 and the mean number of alleles per loci and observed heterozygosity by species and classes is presented in Supplementary Table S3.

**TABLE 3:** Major characteristics of MHC microsatellite markers in a panel of *Camelus bactrianus* and *C. dromedarius*.

Region	Marker	*Camelus bactrianus* (N = 33)				*Camelus dromedarius* (N = 38)			
		N	H_obs_	Number of alleles	PIC	N	H_obs_	Number of alleles	PIC
MHC I	*CAM01*	31	0.7419	11 (364–402)	0.7633	35	0.6000	6 (364–378)	0.7242
MHC I	*CAM02* *^a^*	32	0.6875	9 (296–332)	0.7918	36	0.8333	13 (296–330)	0.8277
MHC I	*CAM03*	33	0.3636	2 (192–194)	0.2999	38	0.2632	2 (192–194)	0.2024
MHC II	*CAM04*	31	0.7742	5 (266–362)	0.5510	34	0.8529	6 (266–370)	0.7523
MHC II	*CAM05*	33	0.5152	4 (274–288)	0.5252	38	0.7632	5 (274–294)	0.6557
MHC II	*CAM06* *^a^*	32	0.5000	4 (141–151)	0.3857	37	0.6342	5 (141–151)	0.5747
MHC II	*CAM07* *^a^*	32	0.6563	4 (296–306)	0.5973	37	0.7027	5 (296–308)	0.6457
MHC III	*CAM08*	27	0.1481	3 (374–382)	0.4165	38	0.1579	4 (374–384)	0.6513
MHC III	*CAM09*	32	0.4063	5 (288–300)	0.5743	37	0.3784	3 (288–300)	0.3123
MHC III	*CAM10*	32	0.7813	10 (374–410)	0.8153	37	0.3514	5 (376–410)	0.2977
MHC III	*CAM11* *^a^*	33	0.5455	5 (242–252)	0.7125	38	0.7895	9 (240–258)	0.7557

^a^ Loci originally identified in the Cornell laboratory.

N—number of animals; Hobs—observed heterozygosity, PIC, Polymorphism Information Content.

Allele frequencies in *Camelus bactrianus* and *C. dromedarius* are shown in Supplementary Table S1. Out of the total number of alleles found in our two groups (79), 63 alleles were observed in the Bactrian camel and 59 in the dromedary. Fifteen alleles unique to the Bactrian camel and 16 unique to the dromedary were observed.

Based on the assumption of independent segregation, it is possible to obtain altogether 9480 haplotypes in the Bactrian camel. Out of them, we were able to identify 27 haplotypes composed of all loci analyzed; three of them occurred with frequencies higher than 0.05: 0.1875, 0.1250 a 0.0833, respectively. For the dromedary, 45 haplotypes out of 8486 were found. No haplotype reached the 0.05 frequency. A complete dataset is in Supplementary Table S2.

## Discussion

Microsatellite markers have been widely used for characterizing the genetic diversity of various human and animal populations (Field and Wills 1996). As unexpressed loci, they are considered to be mostly markers of neutral diversity of the genome (Holderegger et al., 2006). However, microsatellite loci located within complex immunity-related regions may serve for assessing the genetic diversity of these regions, which is assumed to be of adaptive value. As such, especially in combination with analyses of the genetic diversity of expressed loci, they may provide valuable information on the region of interest and may also be used for disease-association studies. For these purposes, we previously characterized the genomic organization of the NKC and LRC regions of camelids, where both expressed loci and microsatellites were identified (Futas et al., 2019).

Identification of polymorphic microsatellite markers in the MHC of the two camel species posed some methodological challenges. Currently, two whole genome assemblies of the dromedary are available, CamDro2 and CamDro3. In this study, CamDro2 was used as the primary resource and all coordinates and corresponding figures refer to this assembly. On the other hand, there is no whole genome assembly currently available for *Camelus bactrianus* whose quality would allow an unequivocal localization of the microsatellites amplified with our primers. Due to differences between the genomes of wild and domestic Bactrian camels (Ming et al., 2020a), the use of the recently published whole genome sequence of the wild Bactrian camel (Ming et al., 2020b) for this purpose is uncertain. However, based on a synteny plot of the camelid MHC region showing its general structural conservation among all camelids (Plasil et al., 2019b), we may assume that the microsatellite loci characterized in this study are markers of the same sub-regions in both domestic species.

Another challenge was the existence of duplicated regions and consequently of duplicated loci reflected by repeated findings of three alleles of some markers, which eventually were eliminated from the definitive panel. Nevertheless, three, four and four markers of the class I, II, and III subregions, respectively, have the potential to be informative for various types of diversity, association and/or evolutionary studies.

122 dinucleotide microsatellites were found in the studied MHC regions. However, the final panel we compiled contains only 11 markers. Based on our previous experience, we eliminated *a priori* mononucleotide microsatellites (uncertain identification of individual alleles), three- or more-nucleotide repetitions (low polymorphism) and irregular microsatellites (not suitable as stable markers). Moreover, other common reasons for discarding a given microsatellite were monomorphism and technical problems during amplification (non-specific fragments, low amplification efficiency, *etc.*), since the aim of the study was to identify potentially good quality markers.

The marker diversity characteristics summarized in Table 3; Supplementary Table S1 were primarily calculated to characterize the marker sets rather than the two species. Although the two cohorts genotyped were composed of randomly selected individuals, they are not necessarily populations representative of their species due to the relatively small numbers of individuals and limited information on their relatedness that could be retrieved from the owners. However, clear interspecific differences in the numbers of alleles and observed heterozygosities (Table 3), as well as in allelic frequencies (Supplementary Table S1), were observed. The lowest observed heterozygosity and at the same time the PIC value were found for the *CAM01* and *CAM10* loci in the Bactrian camel, while in dromedaries, it was in loci *CAM04* and *CAM02*. We are not sure to what extent this difference is due to real interspecific differences and how much it may be affected by the low number of camels analyzed. Taking into account this limitation, “species-specific” alleles were identified in all loci except *CAM06* and *CAM08*. No differences in diversity were found between markers located in different MHC sub-regions; their characteristics seem to be primarily determined by the particular locus regardless of its linkage to expressed genes in the respective sub-region (see Supplementary Table S3).

The *in-silico* haplotype analysis revealed high numbers of inferred haplotypes probably reflecting the relatively long distances between the loci analyzed and weak linkage among them (Supplementary Table S2). This assumption is supported by the lower numbers and higher frequencies of haplotypes specific for the three MHC sub-regions. However, no clearly prevailing haplotypes were observed, and at the species level it was not possible to assign potentially species-characteristic haplotypes.

## Conclusions

Eleven polymorphic microsatellite loci were identified in the three MHC subregions I, II and III of dromedaries and Bactrian camels. The major characteristics of these markers and their polymorphism were assessed. Interspecific differences were observed in the numbers of alleles and in allelic frequencies, as well as in the observed heterozygosity.

These loci may be used as markers to study the adaptive genetic diversity of the MHC region in Old World camels.

## Data Availability

The original contributions presented in the study are included in the article/supplementary materials, further inquiries can be directed to the corresponding author.
