# Supplementary material for: Microsatellite markers of the major histocompatibility complex genomic region of domestic camels
Source: Front Genet. 2022 Oct 24;13:1015288. doi: 10.3389/fgene.2022.1015288 (PMC9638106; doi:10.3389/fgene.2022.1015288)
Supplement: Supplementary file 1 [file Table1.docx]

Supplementary Table 1 Allele frequencies in *Camelus bactrianus* and *C. dromedarius*

| **Marker** | ***Camelus bactrianus* (N=33)** | |  | ***Camelus dromedarius* (N=38)** | |
| --- | --- | --- | --- | --- | --- |
|  | **Allele** | **frequency** |  | **Allele** | **frequency** |
| *CAM01* | 364 | 0.177 |  | 364 | 0.086 |
|  | 366 | 0.032 |  | 366 | 0.300 |
|  | 368 | 0.048 |  | 368 | 0.286 |
|  | 372 | 0.016 |  | 372 | 0.229 |
|  | 374 | 0.371 |  | 374 | 0.071 |
|  | 378 | 0.145 |  | 378 | 0.029 |
|  | 380 | 0.016 |  |  |  |
|  | 382 | 0.032 |  |  |  |
|  | 396 | 0.129 |  |  |  |
|  | 400 | 0.016 |  |  |  |
|  | 402 | 0.016 |  |  |  |
| *CAM02* | 296 | 0.156 |  | 296 | 0.042 |
|  | 302 | 0.156 |  | 302 | 0.167 |
|  | 304 | 0.063 |  | 304 | 0.250 |
|  | 306 | 0.328 |  | 306 | 0.042 |
|  |  |  |  | 308 | 0.042 |
|  |  |  |  | 312 | 0.014 |
|  | 314 | 0.109 |  | 314 | 0.153 |
|  | 318 | 0.109 |  | 316 | 0.028 |
|  |  |  |  | 320 | 0.181 |
|  | 324 | 0.031 |  | 324 | 0.014 |
|  |  |  |  | 326 | 0.014 |
|  | 328 | 0.016 |  | 328 | 0.042 |
|  |  |  |  | 330 | 0.014 |
|  | 332 | 0.031 |  |  |  |
| *CAM03* | 192 | 0.758 |  | 192 | 0.132 |
|  | 194 | 0.242 |  | 194 | 0.868 |
| *CAM04* | 266 | 0.177 |  | 266 | 0.221 |
|  | 340 | 0.129 |  | 340 | 0.059 |
|  | 346 | 0.597 |  | 346 | 0.118 |
|  | 358 | 0.032 |  |  |  |
|  | 362 | 0.065 |  |  |  |
|  |  |  |  | 364 | 0.309 |
|  |  |  |  | 366 | 0.221 |
|  |  |  |  | 370 | 0.074 |
| *CAM05* | 274 | 0.212 |  | 274 | 0.421 |
|  | 276 | 0.015 |  |  |  |
|  |  |  |  | 282 | 0.224 |
|  | 284 | 0.576 |  | 284 | 0.105 |
|  | 288 | 0.197 |  | 288 | 0.013 |
|  |  |  |  | 294 | 0.237 |
| *CAM06* | 141 | 0.172 |  | 141 | 0.243 |
|  |  | 0.000 |  | 145 | 0.014 |
|  | 147 | 0.734 |  | 147 | 0.135 |
|  | 149 | 0.078 |  | 149 | 0.541 |
|  | 151 | 0.016 |  | 151 | 0.068 |
| *CAM07* | 296 | 0.188 |  | 296 | 0.365 |
|  | 300 | 0.141 |  | 300 | 0.014 |
|  | 302 | 0.531 |  | 302 | 0.149 |
|  | 306 | 0.141 |  | 306 | 0.365 |
|  |  |  |  | 308 | 0.108 |
| *CAM08* | 374 | 0.704 |  | 374 | 0.355 |
|  | 380 | 0.148 |  | 380 | 0.355 |
|  | 382 | 0.148 |  | 382 | 0.132 |
|  |  |  |  | 384 | 0.158 |
| *CAM09* | 288 | 0.375 |  | 288 | 0.797 |
|  | 290 | 0.453 |  | 290 | 0.135 |
|  | 294 | 0.016 |  |  |  |
|  | 298 | 0.094 |  |  |  |
|  | 300 | 0.063 |  | 300 | 0.068 |
| *CAM10* | 374 | 0.016 |  |  |  |
|  | 376 | 0.234 |  | 376 | 0.811 |
|  |  |  |  | 378 | 0.014 |
|  | 388 | 0.141 |  |  |  |
|  | 392 | 0.031 |  |  |  |
|  | 396 | 0.031 |  |  |  |
|  | 398 | 0.094 |  |  |  |
|  | 400 | 0.250 |  | 400 | 0.135 |
|  | 404 | 0.109 |  |  |  |
|  | 406 | 0.031 |  | 406 | 0.027 |
|  | 410 | 0.063 |  | 410 | 0.014 |
| *CAM11* |  |  |  | 240 | 0.026 |
|  | 242 | 0.046 |  | 242 | 0.408 |
|  | 246 | 0.182 |  | 246 | 0.053 |
|  | 248 | 0.212 |  | 248 | 0.092 |
|  | 250 | 0.349 |  | 250 | 0.132 |
|  | 252 | 0.212 |  | 252 | 0.105 |
|  |  |  |  | 254 | 0.118 |
|  |  |  |  | 256 | 0.040 |
|  |  |  |  | 258 | 0.026 |
