# Supplementary material for: Microsatellite markers of the major histocompatibility complex genomic region of domestic camels
Source: Front Genet. 2022 Oct 24;13:1015288. doi: 10.3389/fgene.2022.1015288 (PMC9638106; doi:10.3389/fgene.2022.1015288)
Supplement: Supplementary file 2 [file Table2.docx]

Supplementary Table 2 In-silico haplotype analysis

|  |  | **Total number of possible haplotypes** | **Identified haplotypes** | **Most frequent t haplotypes (˃0,05)**  **(first row – names of loci)** | **Frequency** |  |
| --- | --- | --- | --- | --- | --- | --- |
| ***Camelus bactrianus***  **(N=33)** | MHC I, II, and III | 9480 | 27 | CAM01 02 03 04 05 06 07 08 09 10 11  374 306 192 346 284 147 302 374 290 400 250  374 306 192 346 284 147 302 374 290 398 250  364 302 194 362 274 141 306 382 288 376 248 | 0.1875  0.1250  0.0833 |  |
|  | MHC I | 55 | 14 | CAM01 02 03  374 306 192  378 296 192  364 302 194  396 318 192 | 0.3958  0.1458  0.1250  0.0833 |  |
|  | MHC II | 81 | 14 | CAM04 05 06 07  346 284 147 302  362 274 141 306  266 274 147 302  266 284 141 306 | 0.5000  0,0833  0.6250  0.6250 |  |
|  | MHC III | 89 | 22 | CAM08 09 10 11  374 290 400 250  374 290 398 250  374 290 410 250 | 0.2187  0.1250  0.0729 |  |
| ***Camelus dromedarius***  **(N=38)** | MHC I, II, and III | 8486 | 45 | no |  |  |
|  | MHC I | 59 | 20 | CAM01 02 03  372 304 194  368 320 194  366 302 194  368 314 194 | 0.1833  0.1667  0.1167  0.1000 |  |
|  | MHC II | 144 | 22 | CAM04 05 06 07  364 274 149 296  364 274 149 296  364 274 141 306 | 0.1500  0.1459  0.1167 |  |
|  | MHC III | 64 | 27 | CAM08 09 10 11  380 288 376 242  380 288 376 242  382 288 376 242 | 0.1333  0.1167  0.0833 |  |

The calculation was done using software Arlequin ver 3.5 (Excoffier & Lischer, 2010).
