# Supplementary material for: Microsatellite markers of the major histocompatibility complex genomic region of domestic camels
Source: Front Genet. 2022 Oct 24;13:1015288. doi: 10.3389/fgene.2022.1015288 (PMC9638106; doi:10.3389/fgene.2022.1015288)
Supplement: Supplementary file 3 [file Table3.docx]

Supplementary Table 3 Comparison of the basic statistics calculated for *Camelus bactrianus* and *Camelus dromedarius*

|  | **Region** | **Mean number of alleles per locus** | **H*obs*** |
| --- | --- | --- | --- |
| ***Camelus bactrianus***  **(N=33)** | MHC I, II and III | 5.273 | 0.5682 |
|  | MHC I | 6.333 | 0.6111 |
|  | MHC II | 4.000 | 0.6146 |
|  | MHC III | 5.750 | 0.4896 |
| ***Camelus dromedarius***  **(N=38)** | MHC I, II and III | 5.455 | 0.5818 |
|  | MHC I | 7.000 | 0.5556 |
|  | MHC II | 5.000 | 0.7833 |
|  | MHC III | 4.750 | 0.4000 |

N – number of animals; H*obs* – observed heterozygosity for all loci in region
